# Psychological Safety as an Enduring Resource Amid Constraints

**DOI:** 10.3389/ijph.2024.1607332

**Published:** 2024-05-31

**Authors:** Hassina Bahadurzada, Amy Edmondson, Michaela Kerrissey

**Affiliations:** ^1^ Harvard Business School, Boston, MA, United States; ^2^ T. H. Chan School of Public Health, Harvard University, Boston, MA, United States

**Keywords:** psychological safety, burnout, turnover, conservation of resources, crisis management

## Abstract

**Objectives:**

While psychological safety is recognized as valuable in healthcare, its relationship to resource constraints is not well understood. We investigate whether psychological safety mitigates the negative impact of resource constraints on employees.

**Methods:**

Leveraging longitudinal survey data collected from healthcare workers before and during the COVID-19 crisis (*N* = 27,240), we examine how baseline psychological safety relates to employee burnout and intent to stay over time, and then investigate this relationship relative to resource constraints (i.e., the inadequacy of staffing and tools).

**Results:**

Using hierarchical linear models, we find that psychological safety has enduring protective benefits for healthcare workers during periods of stress, and that these benefits mitigate the negative consequences of resource constraints for burnout and turnover intent over time.

**Conclusion:**

These findings extend the empirical basis for psychological safety and suggest that investments in building psychological safety can foster employee resilience and organizational commitment, even when resources are strained.

## Introduction

Since its initial conceptualization, psychological safety—defined by the belief that speaking up will not lead to embarrassment, rejection, or punishment—has become well established in the organizational literature [1, 2]. It has been linked to improving aspects of team behavior, such as information sharing [3] and performance [4], as well as to various aspects of employee wellbeing, such as feelings of vitality [5], engagement and creativity at work [6] and reduced emotional exhaustion and burnout [7]. In the context of healthcare, which often involves multidisciplinary, complex, high stakes work that requires input and engagement from all team members, the concept of psychological safety has been documented as particularly important, both in research [8–10] and practice [11].

The COVID-19 pandemic provided an unfortunate but informative context in which to explore how sudden and intense resource constraints might interact with psychological safety to affect clinician and staff experience at work. The pandemic led to an unprecedented crisis in healthcare characterized first by resource depletion (e.g., lack of personal protective equipment) and over time by increasingly intense staffing shortages as personnel quit or moved to non-acute care settings or administrative positions [12]. Many have talked about the importance of psychological safety during the pandemic [13]. However, research has not yet, to our knowledge, examined whether or how psychological safety prior to the COVID crisis related to feelings of burnout and intent to stay during the crisis, particularly in relation to sudden and strong constraints in staffing and other resources at the time.

In this paper, we conduct longitudinal and cross-sectional analyses to examine how baseline psychological safety as perceived by healthcare workers prior to the pandemic in 2019, and later in 2021, across a large number of departments in a US-based healthcare system, relates to employee burnout and intent to stay in their jobs in 2021—after employees had been through the initial crisis of the COVID-19 pandemic. In addition, we explore how resource and staffing constraints relate to burnout and intent to stay with the organization in 2021. These analyses are followed by a cross-sectional and longitudinal moderation analyses in 2021 to examine if and to what extent psychological safety can mitigate the deleterious relationship of staffing and resource constraints with intent to stay and burnout.

### Conceptual Model and Hypotheses

We draw on the conservation of resources theory [14] to conceptualize how psychological safety relates to employee burnout and intent to stay in an organization during periods of crisis that bring resource depletion and staffing constraints. Burnout is defined as a work-related syndrome consisting of the three elements: emotional exhaustion, depersonalization, and reduced sense of personal accomplishment [15]. In the past, scholars have applied conservation of resources theory to burnout [16, 17]; we build on this to argue that psychological safety is particularly salient during periods of high uncertainty. Conservation of resources theory posits that individuals experience stress when they lose resources [18]. If resource loss is substantial, it can hamper employees’ ability to meet the demands of their jobs and thereby induce stress, which can lead to burnout and employee turnover if the situation persists [19].

#### Psychological Safety as Protective Against Burnout and Turnover During Periods of Stress

Conservation of resources theory defines resources broadly, ranging from objects to the social environment. The social environment, if it increases the chances of obtaining positive reinforcement and social support, can be perceived as resource replenishing [20]. Hence, we hypothesize that a psychologically safe climate, where speaking up is encouraged and addressing errors emphasizes learning rather than interpersonal judgement, can be considered a social resource surplus, offsetting the unexpected material resource loss induced by the COVID-19 pandemic. More specifically, we hypothesize that psychological safety (both when established prior to crisis and when reaffirmed during crisis) is a resource associated with lower burnout and greater organizational commitment.

Hypothesis 1 Psychological safety during crisis is associated with lower levels of burnout (1a) and greater levels of intent to stay (1b).

Hypothesis 2 Psychological safety established prior to crisis is associated with lower levels of burnout (2a) and greater levels of intent to stay (2b) during crisis periods.

#### Resource Constraints as Amplifiers of Burnout and Turnover During Periods of Stress

Conservation of resources theory describes a clear relationship between resources and the stress that employees experience; when resources are suddenly or gradually depleted, individuals experience stress that can lead to burnout and potentially turnover as employees quit. Research indicates that individuals who possess resource surpluses- by engaging in resource replenishing activities- are more likely to experience wellbeing and resilience [21, 22], whereas individuals who do not possess resource surpluses are more vulnerable to experiencing loss spirals through which initial losses cannot be offset by already scarce resources and it becomes increasingly difficult for individuals to recover [23–27]. During the COVID-19 pandemic, resources were constrained for many healthcare delivery organizations, particularly material resources like personal protective equipment and staffing resources (i.e., people quit or fell ill), leading to high levels of vacancies and absenteeism. However, the degree of resource constraints varied across departments, we thus hypothesize that individuals in departments experiencing more acute resource constraints experienced relatively stronger burnout and turnover intentions than their peers in less constrained environments.

Hypothesis 3 Greater level of adequate tools is associated with lower levels of burnout (3a) and greater intent to stay (3b).

Hypothesis 4 Greater level of adequate staffing is associated with lower levels of burnout (4a) and greater intent to stay (4b).

#### Psychological Safety as Moderator of the Relationships Between Resource Constraints and Burnout/Turnover

Healthcare workers are known to have limited resource surpluses; nurses and physicians are faced with time pressure, limited tools, and high stakes, impeding their ability to execute their clinical tasks and engage in resource-replenishing activities that can help them build resource surpluses [28, 29]. In practice, the immediate nature of concrete resources like material and time pressure can give rise to the assumption that focusing on less concrete resources like psychological safety is less important, or would occur at the expense of ensuring concrete resources [30]. This concern became particularly salient as the COVID pandemic intensified the strained conditions under which caregivers were providing care. Returning to conservation of resources theory, however, we suggest an alternate view and hypothesis.

If psychological safety is conceptualized as a social resource, then it is likely to play an interactive role with constrained material and human resources—rather than being substitutive—in lowering the barrier to speaking up about concerns and asking questions. In this way, psychological safety may make it easier for people to ameliorate the material losses they experience (e.g., by voicing a concern about lack of personal protective equipment or a need for days off). If so, psychological safety would mitigate the effects of resource constraints on burnout and turnover. We thus hypothesize that psychological safety beneficially moderates the relationships between resource/staffing constraints and burnout/turnover. We hypothesize these relationships to hold both for psychological safety experienced in the current moment of organizational stress and for psychological safety as experienced prior to the current moment of organizational stress.

Hypothesis 5 Psychological safety (2021) moderates the relationship of adequate tools with burnout (5a) and intent to stay (5b) in 2021.

Hypothesis 6 Psychological safety (2021) moderates the relationship of adequate staffing with burnout (6a) and intent to stay (6b) in 2021.

Hypothesis 7 Baseline psychological safety (2019) moderates the relationship of adequate tools with burnout (7a) and intent to stay (7b) in 2021.

Hypothesis 8 Baseline psychological safety (2019) moderates the relationship of adequate staffing with burnout (8a) and intent to stay (8b) in 2021.

## Methods

### Survey Instrument and Administration

We obtained data from a large, multi-site health system with a main campus in the midwestern region in the United States. The health system administers a bi-annual electronic census survey in English to all employees to examine their perception of their work environment. The data used for this study was collected in May 2019 (*n =* 42,196, response rate 87%) and May 2021 (*n* = 50,471, response rate 80%). The data we obtained was anonymized (i.e., with no individual employee identifiers beyond a randomized identification code). In 2019, the survey included items measuring the perceptions of psychological safety, presence of adequate tools, presence of adequate staffing, and how likely respondents were to stay with their organization if they were offered a similar position elsewhere. In 2021, an item was added to measure the extent to which employees felt burned out from their job. Since psychological safety is a measure of climate within an organization that is experienced by all employees, we included both clinical and non-clinical staff, resulting in an analytic sample of 27,240 respondents, attributed to approximately 2,030 departmental units in distinct locations. Because this study relied on deidentified secondary data, it was deemed as Not Human Subjects Research; institutional review board ethics approval was waived.

### Measures

#### Independent Variables

We use two measures for resource constraints: 1) the presence of adequate tools, and 2) having adequate staffing, given the centrality of labor as a key resource in care delivery. These were assessed by the item “I get the tools and resources I need to provide the best care/service for our patients” and the item “My team/department is adequately staffed,” both on a five-point agreement scale ranging from strongly disagree [1] to strongly agree [5]. To check for collinearity, we examined the correlation between the two items and found *r* = 0.49, and hence used the items as two separate independent variables. To clarify our terminology in this paper, we speak of “resources constraints” as an umbrella term to describe constraints in both tools and staffing; when describing the distinct sub-categories, we use the terms “adequate tools” and “adequate staffing” specifically.

Psychological safety was measured using four items reflecting the extent to which respondents felt safe to speak up and effectively address care delivery and patient safety concerns within their organization, adapted from the original psychological safety scale used by Edmondson (1999). The items include 1) “I can report patient safety mistakes without fear of punishment” 2) “I feel free to raise workplace safety concerns” 3) “Caregivers will freely speak up if they see something that may negatively affect patient care”, and 4) “Caregivers feel free to question the decisions or actions of those with more authority.” All items were measured on a five-point Likert scale ranging from strongly disagree [1] to strongly agree [5]. The Cronbach’s alpha was 0.79 in 2019 and 0.83 in 2021, indicating internal consistency. The measure was operationalized as a mean over these four variables. All items are measured the same in 2019 and in 2021. The 2019 composite measure indicates a respondent’s baseline psychological safety which refers to the respondents’ perception of psychological safety within his/her department prior to entering a period of organizational crisis as induced by the COVID-19 pandemic. The psychological safety composite measure in 2021 measures psychological safety at the same time as the outcome measures are reported.

#### Dependent Variables

We used burnout and intent to stay measured by the items “I do not feel burned out from my work” and “I would stay with this organization if offered a similar position elsewhere” as dependent variables. The dependent variables were measured at the individual level with five answer options corresponding to increasing intent to stay and decreasing experience of burnout due to the positive wording of the latter item. To ease interpretation, we reverse-scored burnout so that an increasing score corresponded to higher levels of burnout as perceived by the respondent. The single-item burnout measure draws on prior studies validating a single item measure of emotional exhaustion, which have found this approach to exhibit strong and consistent associations with clinician dropout, major medical errors, and suicidality, generating support for the use of single item measure of emotional exhaustion for efficiency in healthcare contexts [31, 32]. The intent to stay variable is used as an indicator for future turnover. We checked for the plausibility of this measure as an indicator of real future turnover by examining the percentage of respondents who do not reappear in the data in 2021 after reporting an intent to leave in 2019; we found that about half of those who reported an intent to leave in 2019 do not then respond to the survey in 2021. While we cannot observe directly the reason for later non-response, given the high response rate of the survey overall, this is indicative of a plausible relationship between intending to leave and actually leaving—or at least, being sufficiently disengaged to stop responding to organizational surveys.

#### Control Variables

Control variables for gender, role, race, and tenure were included based on prior literature associating demographic and status characteristics with psychological safety and burnout [10, 33, 34]. Gender was operationalized as a binary variable indicating male/female, with female being the reference category. Role and race were also included as binary variables indicating physician versus other (e.g., nurses, other clinical professionals, and non-clinical professional), and White versus other, respectively. The choice for White as reference category was informed by the race distribution in our sample as the majority of our respondents reported being White. Tenure was collapsed in categories 1–10 years and >10 years.

### Statistical Analyses

For this study, we leveraged organizational survey data in 2019 and 2021, merging the datasets at the individual level and only including respondent for whom we had data in both years. We conducted univariate analysis to examine the mean, standard deviation and distribution of each measure. We computed Cronbach’s alpha to assess the internal consistency of the items measuring psychological safety and explored correlation between the independent and dependent variables to explore potential collinearity (Supplementary Table S1). Our first set of analyses (main effect analyses) examined how psychological safety in 2019 and in 2021 each relate to burnout and intent to stay in 2021. We then explored the association between the perception of adequate tools and adequate staffing in 2021 with intent to stay and burnout in 2021. All models were hierarchical linear regression models accounting for the nested nature of our data: clinical and non-clinical staff are nested within their departments in specific locations. We used linear regression models to ease the interpretation of the models [35, 36].

We conducted moderation analysis examining how psychological safety affects the relationships between resource constraints and burnout/intent to stay in 2021. To examine the resource surplus properties of psychological safety over time, we repeated our moderation analyses using baseline psychological safety in 2019 to examine how psychological safety before the pandemic relates to burnout and intent to stay with the organization in 2021. We graphically visualized the moderating effects and performed simple slope analyses to confirm consistency in direction, magnitude, and significance of the slope between varying levels of psychological safety and resources constraints. The main effect analyses and moderation analyses are reported in separate tables for clarity. All analyses were conducted using STATA version 17.

## Results

Respondent characteristics of the analytic sample are reported in Table 1. Among the 27,240 respondents, 77.56% were female. For role, 34.65% were nurses, 6.27% physicians, 18.23% other clinical professionals, and 40.85% other non-clinical professionals. Over half of the respondents reported a tenure between 1 and 10 years (54.63%), with 0.21% reporting a tenure of less than 1 year and the remaining 45.36% a tenure of more than 10 years. The majority of the respondents were White (78.05%), followed by 12.58% Black or African American, 3.97% Asian, and 3.65% Hispanic respondents.

**TABLE 1:** Sample characteristics (*N* = 27,240; United States, 2021).

Characteristics	*N* (%)
Female*	21,082 (77.56%)
Role	
Physician	1,709 (6.27%)
Nurse (NO, RN, LPN, CRNA, Nursing Asst)	9,439 (34.65%)
Other Clinical Professional	4,965 (18.23%)
Other Non-Clinical Professional	11,127 (40.85%)
Race*	
White (not of Hispanic origin)	21,205 (78.05%)
Black or African American	3,417 (12.58%)
Asian	1,078 (3.97%)
Hispanic or Latino	993 (3.65%)
Other	475 (1.75%)
Tenure	
Less than 1 year	56 (0.21%)
Tenure 1–10 years	14,827 (54.63%)
Tenure >10 years	12,357 (45.36%)

*Female and race do not up to 100% due to missingness in reporting these items.

Table 2 presents descriptive statistics. In 2019, the composite measure for psychological safety with mean 4.15 and standard deviation 0.72, corresponds qualitatively closest to respondents agreeing on the presence of a psychologically safe climate. In 2021, the mean of the composite measure for psychological safety decreased somewhat to 4.11 and the standard deviation increased to 0.77. For the items measuring resource constraints, 75.70% of respondents agreed to having the tools to provide the best care for their patients; 45.56% agreed there was adequate staffing in their team or department. For the outcome variables, 73.08% at least agreed that if offered a similar position elsewhere, they would stay with their organization. The mean for burnout was 2.85, which is close to the scale midpoint and the qualitative answer “neither agree nor disagree.”

**TABLE 2:** Measure descriptives: N, mean, standard deviation (SD), and response distribution (United States, 2019–2021).

				Response distribution (%)				
Measures	N	Mean	SD	1	2	3	4	5
Psychological safety (2019)	27,240	4.15	0.72					
Report patient safety mistakes without fear of punishment	24,731	4.42	0.83	1.59	2.28	6.11	32.11	57.91
Feel free to raise workplace safety concerns	26,868	4.36	0.8	1.06	2.31	7.23	38.31	51.08
Caregivers speak up if something negatively affects patient care	25,604	4.32	0.84	1.23	3.21	7.88	38.19	49.5
Caregivers feel free to question those with more authority	26,680	3.55	1.09	4.62	13.34	23.92	38.21	19.91
Psychological safety (2021)	27,240	4.11	0.77					
Report patient safety mistakes without fear of punishment	24,840	4.39	0.85	1.48	2.72	6.91	32.85	56.04
Feel free to raise workplace safety concerns	26,664	4.3	0.87	1.48	3.41	7.83	38.25	49.03
Caregivers speak up if something negatively affects patient care	25,505	4.28	0.88	1.38	3.76	8.61	37.88	51.64
Caregivers feel free to question those with more authority	26,811	3.52	1.1	5.19	13.65	24.88	36.99	19.3
Burnout	27,096	2.85	1.25	14.91	30.05	22.72	19.98	12.34
Intent to stay	26,887	4.01	0.96	1.82	4.47	20.63	36.63	36.45
Adequate tools and resources	25,964	3.96	0.99	2.52	6.97	14.82	43.15	32.55
Adequate staffing	27,046	3.11	1.33	14.45	22.84	17.14	28.23	17.33

Table 3 presents the main associational analyses. We found support for hypotheses 1a through 2b: psychological safety in the moment (2021) and baseline psychological safety (2019) had a statistically significant protective relationship with burnout (H2a) and related positively with intent to stay in 2021 (H2b). For example, these findings can be interpreted as: a one-point increase in psychological safety relates to a 0.72-point decrease in burnout and 0.63-point increase in intent to stay in 2021. We also found support for hypotheses 3a through 4b: the presence of adequate tools and adequate staffing are related to lower levels of burnout and higher intent to stay, where a one-point increase in the perception of adequate tools and a one-point increase in the perception of adequate staffing led to, respectively, a 0.60-point and 0.50-point decrease in burnout and a 0.50-point and 0.28-point increase in intent to stay.

**TABLE 3:** Hierarchical linear regression models relating psychological safety, adequate tools and resources, and adequate staffing to burnout and intent to stay (United States, 2019–2021).

	Dependent variables							
	Burnout (2021)				Intent to stay (2021)			
	H1a	H2a	H3a	H4a	H1b	H2b	H3b	H4b
Psychological safety (2019)		−0.40**				0.37**		
Psychological safety (2021)	−0.72**				0.63**			
Adequate tools and resources (2021)			−0.60**				0.50**	
Adequate staffing (2021)				−0.50**				0.28**
Controls								
Female	0.08**	0.13**	0.13**	0.12**	0.05**	0.01	0.01	−0.01
Physician	0.15**	0.13**	0.08*	0.08*	−0.04	−0.02	0.02	−0.01
White	0.07**	0.06**	−0.01	0.02	0.05**	0.06**	0.11**	0.09**
Tenure 1–10 years	0.26	0.40*	0.19	0.38**	−0.08	−0.19	−0.02	−0.2
Tenure >10 years	0.12	0.2	0.01	0.26	0.07	0.01	0.15	−0.04
N	27,037	27,037	25,788	26,864	26,828	26,828	25,611	26,660
Department teams	2,036	2,036	2,031	2,036	2,035	2,035	2,030	2,035

* *p* < 0.05; ** *p* < 0.01.

Table 4 displays the moderation analyses, indicating a stable pattern of psychological safety moderating how adequate tools and staffing relate to burnout and intent to stay. In the cross-sectional moderation models, we find support of hypothesis 4a through 5b: Psychological safety significantly moderates how adequate tools and adequate staffing relate to burnout and intent to stay, where a one standard deviation increase in psychological safety leads to a respectively 0.13-point and 0.05-point decrease in burnout and 0.05-point and 0.03-point increase in intent to stay. In the longitudinal models, we see that baseline psychological safety moderates how adequate tools relate to burnout (β = −0.08, *p* < 0.01) and intent to stay (β = 0.03, *p* < 0.01), but the finding is not statistically significant for staffing in that time period. Where significant moderation effects were found, simple slope test confirmed consistency in direction, magnitude, and significance of the slope between varying levels of psychological safety and resources constraints.

**TABLE 4:** Hierarchical linear moderation models relating psychological safety, adequate tools and resources, and adequate staffing to burnout and intent to stay (United States, 2019–2021).

	Dependent variables							
	Burnout (2021)				Intent to stay (2021)			
	H5a	H6a	H7a	H8a	H5b	H6b	H7b	H8b
Psychological safety (2019)			−0.17**	−0.21**			0.18**	0.28**
Psychological safety (2021)	−0.48**	−0.48**			0.41**	0.54**		
Adequate tools and resources (2021)	−0.43**		−0.57**		0.33**		0.46**	
Adequate staffing (2021)		−0.38**		−0.47**		0.14**		0.24**
Psych safety × adeq tools	−0.13**		−0.08**		0.05**		0.03**	
Psych safety × adeq staffing		−0.05**		0.01		0.03**		−0.01
Controls								
Female	0.08**	0.07**	0.11**	0.10**	0.05**	0.05**	0.03**	0.03
Physician	0.10**	0.09**	0.07*	0.07*	−0.00	−0.03	0.02	−0.00
White	0.03	0.04**	0.01	0.03	0.08**	0.06**	0.10**	0.07**
Tenure 1–10 years	0.16	0.27*	0.19	0.36**	−0.00	−0.09	−0.02	−0.18
Tenure >10 years	0.02	0.18	0.02	0.25	0.14	0.04	0.15	−0.03
*N*	25,788	26,864	25,788	26,864	25,611	26,660	25,611	26,660
Department teams	2,031	2,036	2,031	2,036	2,030	2,035	2,030	2,035

* *p* < 0.05; ** *p* < 0.01.

Across all models in Tables 3, 4, female respondents and respondents who identify as physician report higher levels of burnout compared to male respondents and respondents in other professions. Additionally, respondents identifying as White report higher intent to remain with the organization, compared to respondents who do not identify as White.

**FIGURE 1:**
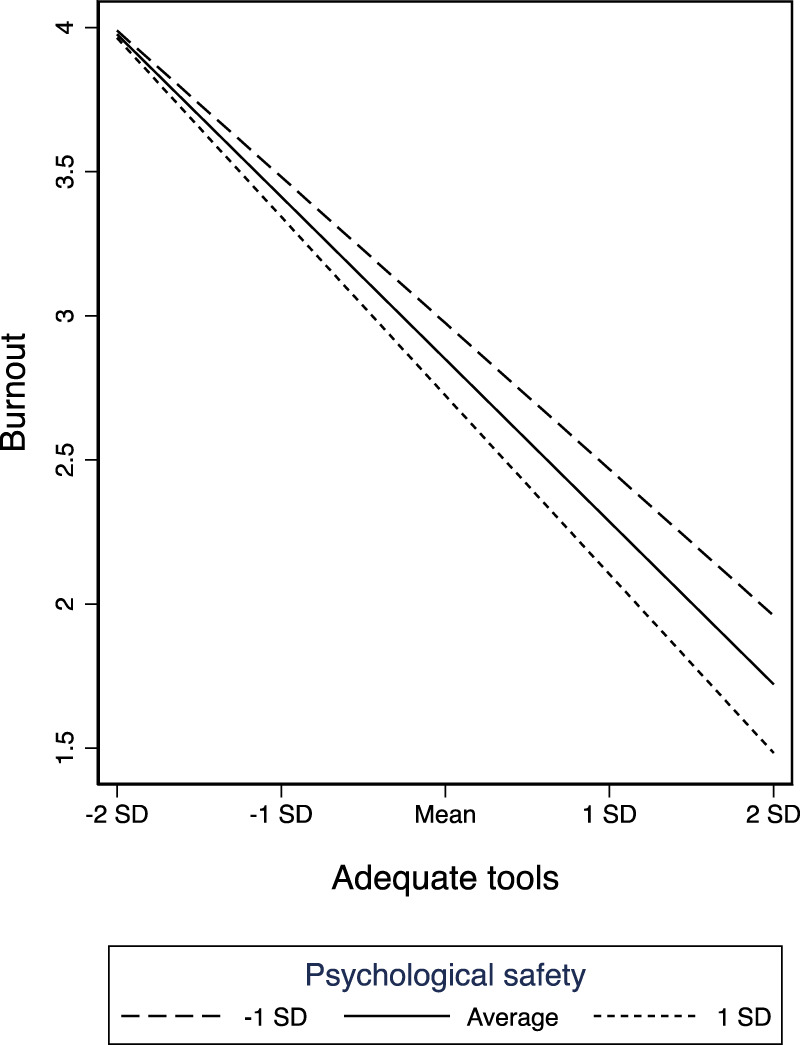
Baseline psychological safety moderating how adequate tools relate to burnout (United States, 2019–2021).

**FIGURE 2:**
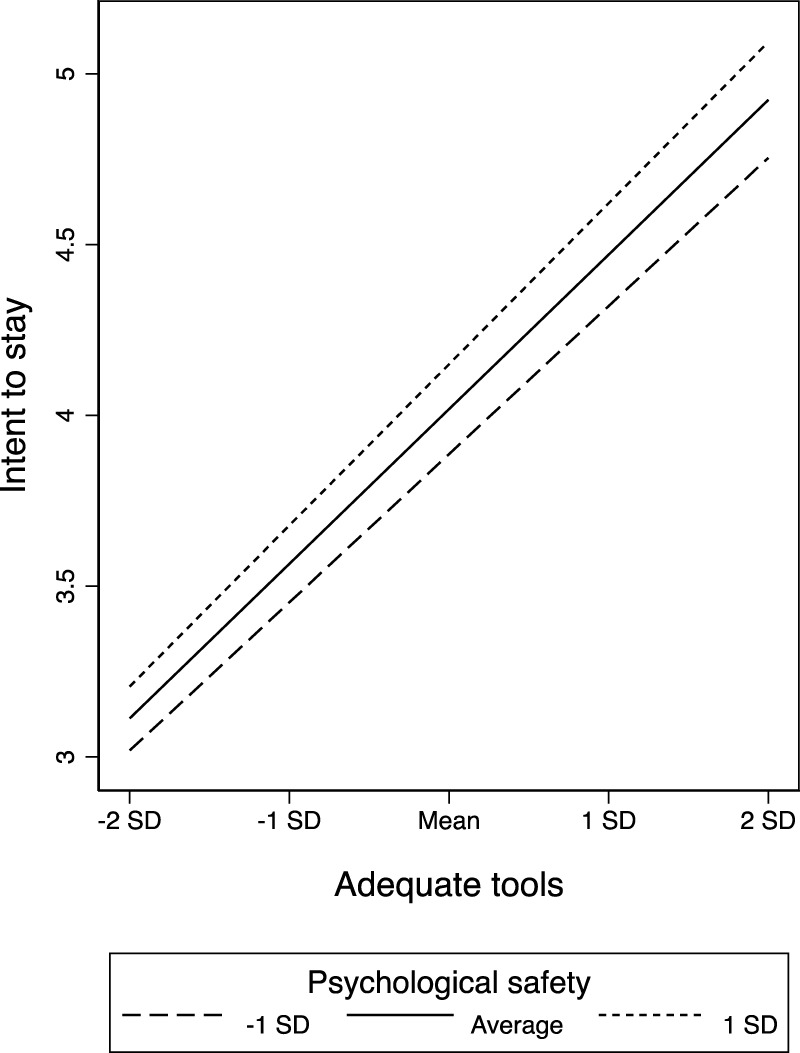
Baseline psychological safety moderating how adequate tools relate to intent to stay (United States, 2019–2021).

Figure 1 graphically depicts the relationship between adequate tools and burnout as moderated by baseline psychological safety. It shows how psychological safety interacts with having adequate tools to reduce levels of burnout across levels of resources, though the interactive benefit appears to diminish at extremely low resource levels (about two standard deviations below the mean). Figure 2 depicts psychological safety’s moderating relationship for adequate tools using intent to stay (rather than burnout) as the outcome. We do not graphically present the moderation between adequate staffing and the outcomes since these findings were not statistically significant (the visual pattern is similar).

## Discussion

Amid interest in how to protect healthcare workers’ wellbeing and reduce turnover, our longitudinal study identifies psychological safety as a key resource with sustained benefit during a period of high stress and constrained resources. We found that psychological safety prior to COVID-19 offered a protective benefit for healthcare workers well into the pandemic. These benefits mitigated the negative consequences of resource and staffing constraints for burnout and turnover intent.

While psychological safety is widely accepted as an important aspect of a safety climate in healthcare [2], its longitudinal impact and importance in retaining and protecting employees from burnout amidst organizational crisis has received limited attention. Incorporating time through a longitudinal research design, our results support an enduring protective association between the presence of high psychological safety and employee wellbeing and intent to stay with the organization. Our finding that psychological safety acts as a source of continued individual resilience amid straining circumstances is consistent with the idea that positive relationships and social support are valued social resources in organizations [20]. This finding is particularly salient given that we studied the longitudinal protective properties of psychological safety over a period of crisis that was characterized by intensification of resource shortages and staffing constraints in an already challenging setting [12].

Despite resource constraints weighing heavily on employees, their commitment to the organization appears to remain higher in a psychologically safe environment. Employees value and prioritize working in an environment where they feel respected and part of a team that focuses on addressing errors to enhance care delivery rather than judging each other [10, 37]. Instability and high turn-over can impede the benefits of social systems in organizations [38]. In these periods, creating a psychologically safe environment helps assure patient and workplace safety while enhancing effective teamwork [10]. Our findings support Edmondson’s (1999) reasoning that for teams facing uncertainty, the gain from engaging in learning behavior to enhance coordination and collaboration can offset the risk of wasting time.

In alignment with conservation of resources theory, which explains how persistent resource constraints can lead to burnout, our analysis suggests that inadequate tools and staffing are associated with higher burnout and greater likelihood of intending to leave the organization. More importantly, our findings echo earlier research indicating that individuals who possess resource surpluses are more likely to experience wellbeing and be more resilient in the face of resource loss, whereas individuals who do not possess resource surpluses are more vulnerable to experiencing loss spirals, impeding their resilience when faced with adversity [23–27].

Our moderation analyses indicate that high psychological safety can ameliorate the effects of the loss of material resources even at highly constrained resource levels—with the benefits diminishing only in the far tail of the distribution. This suggests that there is perhaps a threshold of material resource constraint after which psychological safety may no longer yield a multiplicative positive impact—but that threshold appears to be at the extreme end of resource constraint, below the lowest 2.5th percentile. For those units below this threshold, it may be that rapid practical intervention for improving concrete material resources is vital and no amount of psychological safety will help substantially. For all other units, however, even those that are well below the mean in resources, it appears that psychological safety plays an important role. Hence, even amid organizational crises with other acutely pressing constraints, psychological safety appears to offer a critical resource to help keep employees committed to the organization and mitigate burnout. We note that the moderation finding for staffing constraints, unlike resource constraints, was not statistically significant (though the directionality was similar across the two moderators). It may be that psychological safety going into a crisis helps employees be creative in addressing material challenges but is not sufficient for reducing burnout if employees are extremely overworked and fatigued.

This study has limitations. First, despite the longitudinal nature of our data, which allows inclusion of independent and dependent variables measured in different years, we can only report associations, without causality. Second, we rely on self-report for all variables, which carries the possibility of bias, though having measures across different time points reduces the same-source bias concerns present in the cross-sectional models. Third, we were only able to examine one large health system with a relatively high level of psychological safety (mean: 4.15); this has implications for generalizability and requires future efforts to replicate these analyses elsewhere, particularly organizations with overall lower psychological safety or other important cultural differences. Finally, we used hierarchical linear regression models to ease the interpretation of our results. While there is support for the appropriateness of using linear models with ordinal survey data [35, 36], it is important to note that by applying linear regression models, the underlying assumption is that the distance between the response categories is equal (i.e., the distance between “Strongly disagree” and “Disagree” is equal to the distance between “Disagree” and “Neither agree nor disagree”).

### Conclusion- Implications for Practice and Future Research

Our findings make two primary contributions. First, leveraging longitudinal data we find that baseline psychological safety (prior to crisis) is associated with reductions in burnout and increased intent to stay in an organization undergoing a major disruption. This is a notable contribution, as the majority of the studies on psychological safety in the healthcare setting are cross-sectional [39]. Second, we find that psychological safety can mitigate the negative implications of staffing and resource constraints for burnout and intent to stay.

These results underscore the practical implications of our study, suggesting that psychological safety serves as a fundamental resource in retaining employees and protecting them against burnout. For healthcare organizations, this suggests that investing in efforts to spur psychological safety—well before moments of crisis and amidst crisis that bring on staffing shortages and other resource constraints—may help to establish resilience. For example, as healthcare organizations actively invest in emergency management systems, there may be untapped benefit in considering psychological safety as part of what emergency preparedness planning and emergency management systems address, measure and promote.

Periods of crisis, stress and material constraints are increasingly common in healthcare. Our findings suggest that future research on the interpersonal aspects of enduring and being resilient to these challenges is well warranted. For example, research drawing on multiple methods is needed to examine how employees’ perceptions of the interpersonal climate evolve as constraints and uncertainty increase during more typical periods of staffing and resource constraints beyond the COVID-19 pandemic. For employee and patient wellbeing, an applied and comprehensive understanding of the interpersonal climate and its evolvement in real healthcare work contexts is needed.
