# Supplementary material for: Psychological Safety as an Enduring Resource Amid Constraints
Source: Int J Public Health. 2024 May 31;69:1607332. doi: 10.3389/ijph.2024.1607332 (PMC11176475; doi:10.3389/ijph.2024.1607332)
Supplement: Supplementary file 1 [file DataSheet1.PDF]

Table 1. Measure Descriptives: Alpha and Correlations (United States, 2019-2021)

Measures	Alpha	1	2	3	4	5	6
1. Psychological safety (2019)	0.79	1					
2. Psychological safety (2021)	0.83	0.49**	1				
3. Burnout	n/a	-0.25**	-0.47**	1			
4. Intent to stay	n/a	0.29**	0.52**	-0.48**	1		
5. Adequate tools and resources	n/a	0.34**	0.59**	-0.50**	0.53**	1	
6. Adequate staffing	n/a	0.24**	0.44**	-0.55**	0.40**	0.49**	1

** = p<0.01
